# Kinetics Analysis and ADRC-Based Controller for a String-Driven Vascular Intervention Surgical Robotic System

**DOI:** 10.3390/mi13050770

**Published:** 2022-05-13

**Authors:** Wei Zhou, Shuxiang Guo, Jin Guo, Zhengyang Chen, Fanxu Meng

**Affiliations:** 1 School of Life Science, Beijing Institute of Technology, Beijing 100081, China; zhouwei@bit.edu.cn (W.Z.); chenzhengyang@bit.edu.cn (Z.C.); mengfanxu@bit.edu.cn (F.M.); 2 Key Laboratory of Convergence Medical Engineering System and Healthcare Technology, Ministry of Industry and Information Technology, School of Life Science, Beijing Institute of Technology, Beijing 100081, China

**Keywords:** string-driven slave manipulator, kinetics analysis and ADRC-based controller, vascular intervention surgery, master–slave robotic system, surgeon’s habits

## Abstract

Vascular interventional surgery is a typical method for diagnosing and treating cardio-cerebrovascular diseases. However, a surgeon is exposed to significant X-radiation exposure when the operation is conducted for a long period of time. A vascular intervention surgical robotic system for assisting the surgeon is a promising approach to address the aforementioned issue. When developing the robotic system, a high displacement accuracy is crucial, and this can aid in enhancing operating efficiency and safety. In this study, a novel kinetics analysis and active disturbance rejection control (ADRC)-based controller is proposed to provide high accuracy for a string-driven robotic system. In this controller, kinetics analysis is initially used to improve the accuracy affected by the inner factors of the slave manipulator. Then, the ADRC controller is used to further improve the operating accuracy of the robotic system. Finally, the proposed controller is evaluated by conducting experiments on a vascular model. The results indicate maximum steady errors of 0.45 mm and 6.67°. The experimental results demonstrate that the proposed controller can satisfy the safety requirements of the string-driven robotic system.

## 1. Introduction

Cardio-cerebrovascular diseases are characterized by rapid onset and high lethality. Vascular intervention surgery (VIS) is the main method for dealing with these diseases owing to its advantages, namely fewer postoperative complications and lower trauma and blood loss [1]. However, VIS is risky for the surgeon due to their exposure to X-radiation when performing digital subtraction angiography (DSA), which significantly increases the probability of cancer [2]. Although a heavy lead cloth provides radiation protection to the surgeon, it is prone to causing spondylopathy for the surgeon due to its excessive weight [3]. Additionally, in VIS, intravascular procedures are based on invasive radiology.

With developments in robot technology, a master–slave robotic system is a promising method for protecting surgeons from health threats. Additionally, several systems have been granted FDA approval certificates and are commercialized. In the CorPath^®^ GRX Robot System (Corindus Robotics Inc., Waltham, MA, USA) [4], the master manipulators are joysticks and a touch screen, which are used to control the slave manipulator. In the Amigo Robot System (Catheter Precision Inc., Ledgewood, NJ, USA) [5], a handle without force perception is used as the master manipulator to control the slave manipulator. However, the master manipulators in the CorPath and Amigo robotic systems are not consistent with surgeons’ habits, and the control methods are not presented. The Sensei Robotic System (Hansen Medical Inc., Mountain View, CA, USA) [6] and Genesis RMN System (Stereotaxis Inc., St. Louis, MO, USA) [7] adopted active catheters to implement surgeries, and the active catheters are controlled via a string and a magnetic field. In addition, Hansen Medical was acquired by Auris in 2017, and the technology of the Sensei Robotic System was integrated into the Monarch endovascular robot [8].

Apart from these commercial systems, many scholars have also been researching various VIS robotic systems. Shi et al. developed a master–slave VIS robotic system [9] in which the master manipulator is consistent with surgeons’ habits during interventional surgery. In this master manipulator, the catheter was directly operated, and a contactless displacement measurement method was adopted. In a similar manner, a contactless method was also used in [10]; however, the master manipulator was inconvenient due to the large size of the magnetic field generator. Bao et al. used two commercial devices (Touch X, Geomagic, Andover, MA, USA) as the master manipulator in their proposed system [11,12]. Although this system was calibrated using animal and clinical trials, the master manipulator could not simulate a surgeon’s operating habits during clinical operations, and this increased the time required for learning how to use the new device. Similarly, Omega 3 was used as the master manipulator in a remote-controlled robotic system with multiple functions in [13]. Omisore et al. proposed an automated driving robotic system to operate the catheter. This system used a neuro-fuzzy module to predict and eliminate the backlash behavior based on bounded motion signals [14]. Woo et al. proposed a master–slave robotic system with a novel steerable catheter. This catheter can quickly reduce the operating time and guarantee surgical safety when selecting the target vascular branch [15]. Similarly, Kang et al. proposed a hydraulically steerable guidewire with a diameter of 400 µm, which can realize two different curvatures via a flexible eccentric tube [16]. In general, the control methods used in these systems were not presented in detail in these studies. However, the control system is an important part of master–slave robotic systems.

Moreover, some efforts have been dedicated toward control methods for VIS robotic systems. Hu et al. used a generalized predictive control (GPC) method to improve the performance of robot-assisted cardiovascular surgery [17]. This method can suppress the effects of time-varying delay and parameter identification errors using GPC. Moreover, a terminal sliding mode controller was utilized to improve the system’s robustness. Guo et al. proposed a robust control algorithm to reduce the maximum displacement error to 0.5199 mm [18]. Sankaran et al. proposed a teleoperation endovascular robotic system using a PID method to realize master–slave position control [19], and the operating error in translation was bounded within ±12.5 mm. Yang et al. adopted a fuzzy PID control method to improve tracking accuracy [20], and the maximal error of the system was close to 2 mm. Furthermore, Yang et al. examined the cloud data-based method to realize master–slave remote control [21]. Yan et al. proposed a human–machine collaborative control strategy in a novel master–slave robotic system, which can perceive the insertion state of the guidewire of the operation [22]. The master–slave system’s tracking accuracy exceeds 2 mm. However, the large displacement error is dangerous for patients, according to [23]. Haidegger et al. proposed a force-based control algorithm that provides higher-quality human–machine interaction and utilizes a stochastic approach to improve the precision of integrated setups in surgical robotics [24,25], as well as a cascade control structure using a PID–fuzzy controller to reduce signal latency and improve the control system’s performance [26]. These methods increased the safety and reliability of operation, eased the surgeon’s task, and potentially reduced operating time.

In recent studies on the control methods used in VISRS, PID controllers were the most commonly applied method. Additionally, although the control method is a key issue for improving the displacement accuracy of a robotic system, the related inner factors of the slave manipulator should also be considered for improving control accuracy.

The key contribution of this study corresponds to a novel kinetics analysis and ADRC controller for a master–slave vascular interventional robot. Using the proposed controller, first, the inner systematic error of the slave manipulator is reduced via kinetics analysis, and then the ADRC control method is used to further improve the operating stability and accuracy. The remainder of this paper is organized as follows: The master–slave VIS robotic system is demonstrated in Section 2. In Section 3, the kinetics analysis for the slave manipulator is demonstrated, and the closed-loop control method based on the ADRC controller is introduced. In Section 4, the displacement performance of the robotic system that uses the proposed method is experimentally evaluated. In Section 5, the experimental results are discussed. In Section 6, the conclusions and proposed future work with respect to this study are presented.

## 2. Overview of the Master–Slave Robotic System

**Figure 1:**
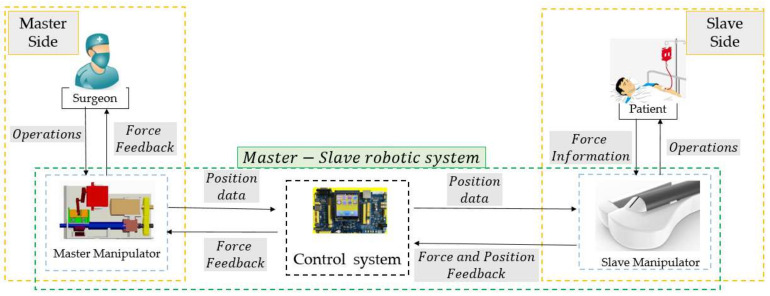
Workflow of the Master–Slave Robotic System.

Figure 1 presents an overview of the master–slave robotic system, which consists of a master manipulator, slave manipulator, and control system. The master manipulator, which is operated by the surgeon, is mounted on a certain location that is free of X-radiation. The slave manipulator is mounted on the operation table to operate the guidewire/catheter from the intervention position to the target location. The movements of the slave manipulator follow the displacement commands generated by the master manipulator via the control system. In addition, the force and position information measured by the slave manipulator are provided as feedback to assist the surgeon in operating smoothly. 

### 2.1. Surgeons’ Habits-Based Master Manipulator

**Figure 2:**
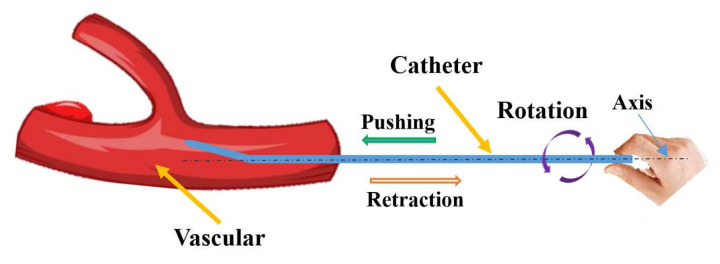
Operating habits of a surgeon.

Surgeons’ habits involve operating the guidewire/catheter to perform three actions (pushing, retraction, and rotation) along the axial direction, as shown in Figure 2. Additionally, by combining different actions, the guidewire/catheter is operated from the intervention position to the target position in a traditional VIS [27]. The functions of the actions are as follows: (1) Pushing and retraction are used to advance and pull back the guidewire/catheter in the vascular tissue; (2) Rotation is used to change the direction of the guidewire/catheter in the vascular tissue; (3) A combination of pushing and rotation is used to position the guidewire/catheter accurately toward the target position, i.e., the location of the lesion.

**Figure 3:**
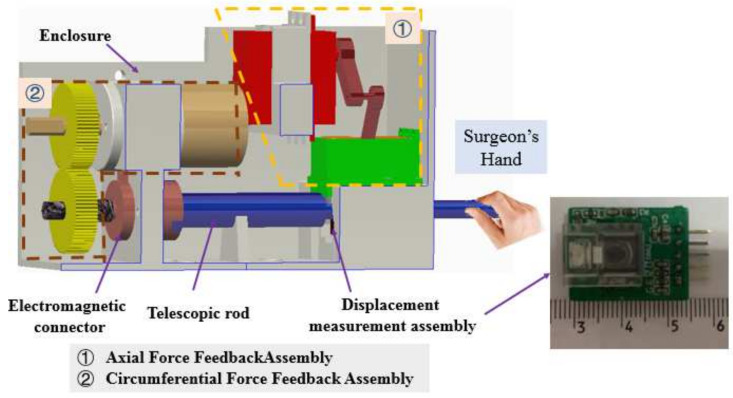
Virtual prototype of the master manipulator.

In the proposed VIS robotic system, the master manipulator is designed based on surgeons’ habits, as shown in Figure 3. The proposed master manipulator consists of a force feedback assembly, displacement measurement assembly, telescopic rod, electromagnetic connector, and enclosure, and it simulates the function and structure of the surgeons’ habits. Furthermore, axial and circumferential force feedback assemblies are used to provide axial and circumferential force feedback, respectively. Axial and circumferential force feedback are realized using a rigid–flexible coupling structure and DC motor via gears, respectively [28]. Moreover, the displacement measurement assembly, which is developed based on an optical mouse sensor (PAW3515DB-TJZA, PixArt Imaging Inc., Taiwan, China), exhibits the ability to simultaneously measure linear and rotary displacement. In addition, the surgeon directly operates the telescopic rod to generate the displacement order of the movement, which is used to control the slave manipulator in real time. The calibration and evaluation experiments for the displacement measurement assembly were presented in [29], and the relationship between the displacement and number of pixels is as follows:

(1) $$L_{dis}=-3*10^{-9}*P_{dis}{}^{3}+5*10^{-6}*P_{dis}{}^{2}+0.032*P_{dis}+1.0568$$

where $L_{dis}$ is the displacement, and $P_{dis}$ is the number of pixels.

### 2.2. Slave Manipulator with a Multi-Slider Structure

**Figure 4:**
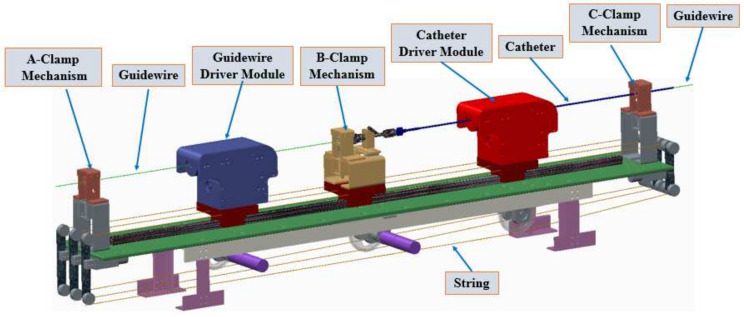
Structure of the slave manipulator.

Figure 4 presents the slave manipulator used in the proposed VIS robotic system, wherein a string-driven method was adopted to connect the module driven by the motor. The slave manipulator employed a multi-slider structure to cooperatively operate the catheter/guidewire, involving a catheter driver module, a guidewire driver module, and three clamp mechanisms. The utilization of the string-driven structure is preferable in our case because the catheter driver module, guidewire driver module, and B clamp mechanism must be coaxial during operations. The catheter and guidewire driver modules, which can clamp the catheter and guidewire without damage, were used to control the movement of the catheter and guidewire, respectively. Based on the cooperation of the catheter driver module, guidewire driver module, and three clamp mechanisms, the collaborative movement function of the guidewire and catheter can be realized. Moreover, the efficiency and performance of this manipulator were evaluated in [11], which demonstrated that this slave manipulator can effectively operate the catheter/guidewire to reach the target position.

## 3. Kinetics & ADRC-Based Controller to Improve the Displacement Accuracy

This section discusses kinetics analysis and the ADRC-based controller, which was utilized to improve the displacement precision of the proposed VIS robotic system. In this method, internal and external factors of the system, which affect the displacement accuracy, were considered. The kinetics analysis method was used to analyze the internal factors, which are related to the mechanical relationships among the transmission parts of the slave manipulator. The internal factors included frictional forces between the string and pulley, driven force, frictional forces between the string and catheter/guidewire driven modules, and inertial force. The external factors affected the master–slave control accuracy of the system, which was further adjusted via the ADRC-based controller.

### 3.1. Kinetics Analysis for the Internal Factors of the Slave Manipulator

**Figure 5:**
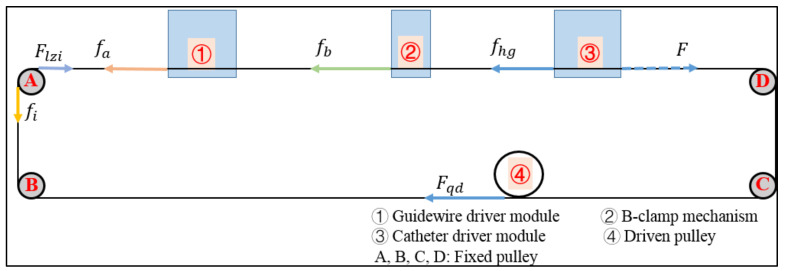
Force analysis diagram of the slave manipulator.

In this section, movement states, namely uniform and accelerated states, of the slave manipulator are analyzed using the kinetics analysis method. Moreover, the purpose of the kinetics analysis is to obtain a more accurate displacement model to minimize the effects of the internal factors of the slave manipulator. Furthermore, the catheter, guidewire driver module, and B-clamp mechanism are driven by the motor (525506, Maxon Motor, Obwalden, Switzerland) using the string via the pulley. The force analysis diagram of the slave manipulator is presented in Figure 5.

The kinetics analysis method is based on Newton’s second law, which states the relationship between force and mass and acceleration as follows:

(2) $$F=m{\ddot{x}}_{out}$$

where m and ${\ddot{x}}_{out}$ denote the mass and acceleration of the catheter driver module, respectively;  F denotes the resultant force, which is a function of $F_{qd},\;f_{hg},\;\;f_{a},\;\;f_{b},\;\;f_{i},\;\mathrm{and}\;F_{lzi}$, and this can be demonstrated as follows:

(3) $$F=Q\left(F_{qd},\;f_{hg},\;\;f_{a},\;\;f_{b},\;\;f_{i},\;F_{lzi}\right)$$

where $F_{qd}$ denotes the driven force generated by the motor via the driven pulley, and $f_{hg}$ denotes the friction between the catheter driver module and leading rail. $f_{a}$ and $f_{b}$ denote the friction generated by the catheter driver module and B-clamp mechanism passing through the string, respectively. Additionally, $f_{i}$ denotes the friction between the string and fixed pulley, and $\;F_{lzi}$ denotes the inertial force of the fixed pulley. It should be noted that $F,\;\;F_{qd},\;f_{hg},\;\;f_{a},\;\;f_{b},\;\;f_{i},\;\mathrm{and}\;F_{lzi}$ are vectors.

The derivation process of the kinetics of the slave manipulator has been demonstrated in [30]. The improved real displacements of the catheter and guidewire driver module and B-clamp mechanism in the slave manipulator are calculated as follows.

(4) $$Dis_{out}=\left\{\begin{array}{c} x_{in}+Dis_{e1},\;\;uniform\;state\\ \iint {\ddot{x}}_{out}dt^{2}-Dis_{e2},\;\;\;\;accelerated\;state\; \end{array}\right.$$

Here, $Dis_{e1}$ and $Dis_{e2}$ denote the compensatory displacements in the uniform and accelerated states, respectively; $x_{in}$ denotes the input displacement. $Dis_{e1}$ and $Dis_{e2}$ are calculated using Equations (5) and (6), respectively, as follows.

(5) $$Dis_{e1}=\frac{0.0411\times x_{in}{}^{2}}{2500}+\frac{0.7456\times x_{in}}{50}-2.731$$

(6) $$Dis_{e2}=-0.0232\times x_{in}{}^{2}+0.2356\times x_{in}+0.7864$$

### 3.2. ADRC-Based Closed-Loop Control Method

**Figure 6:**
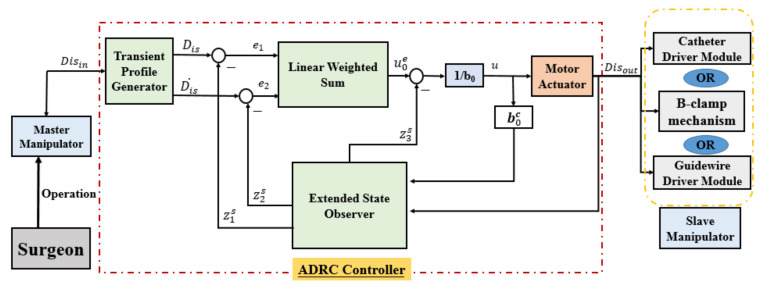
Diagram of the ADRC controller used in the robot-assisted system.

To further ensure the displacement accuracy and operating stability of the slave manipulator, a closed-loop control method is presented based on the ADRC controller shown in Figure 6. The smooth acceleration and deceleration characteristics can aid in the operational safety of the slave manipulator in the VIS robotic system. Moreover, when compared with a proportional–integral–derivative (PID) controller, the ADRC controller exhibits better stability and higher accuracy [29]. The ADRC controller, updated from the PID controller, was developed and applied by Professor Han [31]. The ADRC-based controller [29] consists of a transient profile generator, extended state observer, linear error feedback controller, and compensation controller, which are described in Equations (7)–(10), respectively, as follows:

(7) $$\left\{\begin{array}{c} e=D_{is}-Dis_{in}\\ fh=fhan\left(e,Dis_{2},r_{0},h\right)\\ D_{is}=D_{is}+h\dot{D_{is}}\\ \dot{D_{is}}=\dot{D_{is}}+hfh \end{array}\right.$$

(8) $$\left\{\begin{array}{c} e=z_{1}-Dis_{out},fe=fal(e,\alpha_{1},\delta ),fe_{1}=fal(e,\alpha_{2},\delta )\\ z_{1}^{s}=z_{1}^{s}+h(z_{2}^{s}-3\omega_{0}e)\\ z_{2}^{s}=z_{2}^{s}+h(z_{3}^{s}-3{\omega_{0}}^{2}fe+b_{0}u)\\ z_{3}^{s}=z_{3}^{s}+h(-{\omega_{0}}^{3}fe_{1}) \end{array}\right.$$

(9) $$\left\{\begin{array}{c} \;e_{1}=D_{is}-z_{1}^{s},e_{2}=\dot{D_{is}}-z_{2}^{s}\\ u_{0}=k_{1}\times e_{1}+k_{2}\times e_{2} \end{array}\right.$$

(10) $$u=u_{0}^{e}-\frac{z_{3}\left(t\right)}{b_{0}^{c}}$$

where $Dis_{in}$ denotes the reference value, which is the input value of the ADRC-based controller. $D_{is}$ denotes the transient value, $\dot{D_{is}}\;$ denotes the differential value of $D_{is}$. $D_{is}\;$, and $\dot{D_{is}}\;$ denotes the displacement and movement velocity of this controller, respectively; fh denotes the maximum speed synthesis function; $r_{0}$ denotes the speed factor; h denotes the filter factor. $r_{0}$ is used to control the tracking speed, and h can be used to filter the jitter. Furthermore, $Dis_{out}$ denotes the output value; $z_{1}^{s}$, $z_{2}^{s}$, and $z_{3}^{s}$ denote the status observer values of $D_{is}$, $\dot{D_{is}}$, and the total disturbance of the system (including the internal disturbance and the external disturbance), respectively; $\omega_{0}$ denotes the bandwidth of the state observer; $b_{0}^{c}$ denotes the compensating parameter; $u_{0}^{e}$ denotes the error feedback controlled plant; $k_{1}$ and $k_{2}$ denote the coefficient of the error and the error derivatives, respectively.

## 4. Experiments and Results

### 4.1. Comparative Experiments

**Figure 7:**
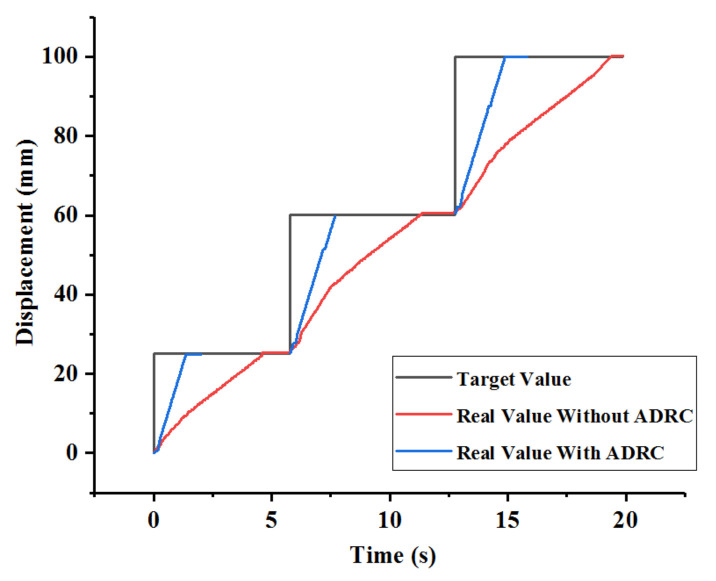
Results of comparative experiments.

This section discusses experiments conducted to compare the performance of two methods. One experiment only involved kinetics analysis, which was utilized to reduce systematic errors. The other is the proposed method in this study, which utilized the ADRC controller based on the kinetics analysis. Therefore, the difference between these two methods is whether the ADRC controller is utilized or not. The experimental method involves providing target displacements (25 mm, 35 mm, and 40 mm) using the serial port assistant and using the two methods for controlling the catheter driver module in the slave manipulator to follow target displacements. The results are presented in Figure 7. As shown, the modifying times of the controller with ADRC for the aforementioned displacements are 1.98 s, 1.89 s, and 3.03 s. The modifying times of the controller without ADRC are 4.59 s, 5.58 s, and 6.60 s for the aforementioned displacements. Therefore, the controller with ADRC is better than that without ADRC in terms of response speed. Additionally, the maximum errors are 0.64 mm and 0.25 mm when using the controller without ADRC and the controller with ADRC, respectively. This shows that the controller with ADRC exhibits higher accuracy. Additionally, the overshoot phenomenon exists in the controller without ADRC, and the maximum value is 0.64 mm. This can potentially pose a risk during surgery. In general, the comparative experiments show that the controller with ADRC exhibits the ability to improve the operating accuracy of the catheter driver module.

### 4.2. Evaluation Experiments

**Figure 8:**
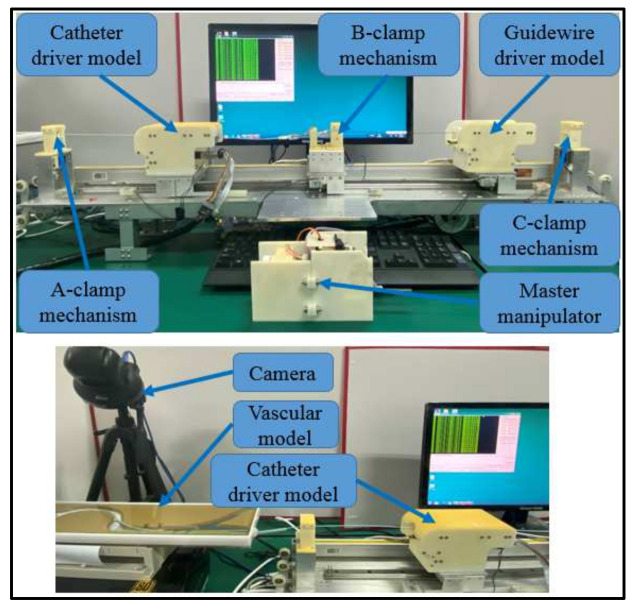
Experimental setup for the evaluation experiments.

**Figure 9:**
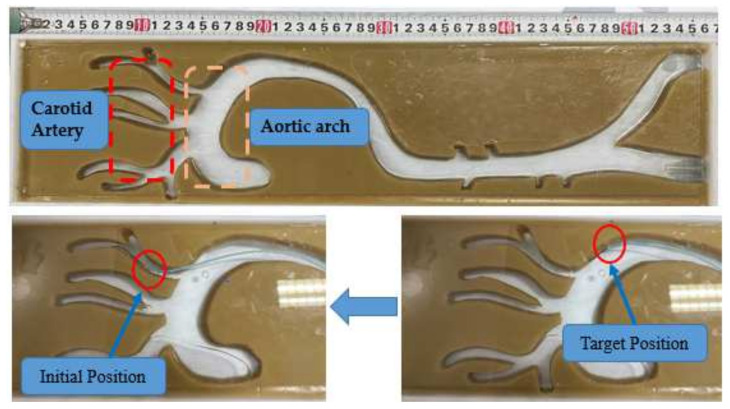
Vascular model in the experiments.

(1) Experimental Method: The experimental setup, which includes the slave manipulator, master manipulator, catheter, camera, and two-dimensional vascular model, for the evaluation experiments is presented in Figure 8. Furthermore, the vascular model is manufactured using paraffin wax, as shown in Figure 9, in which the initial and target positions in the experiments are marked. This vascular model was used in [32] to estimate the operational skills of a surgeon, which demonstrates the efficiency of the vascular model for research.

**Figure 10:**
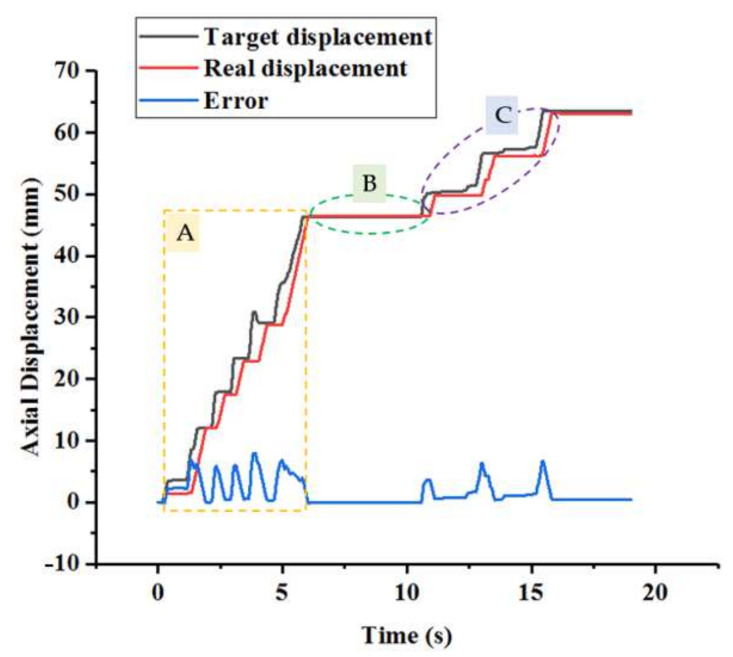
Axial displacements during the experiments.

The operator operates the master manipulator to generate displacements, which include axial and circumferential displacements, assisted by the camera. Furthermore, the displacement control of the proposed robotic system adopts a closed-loop control method using the kinetics analysis and ADRC-based controller. Additionally, in the proposed robotic system, the $\omega_{0}$, $k_{1}$, $k_{2}$, and $b_{0}$ parameters of the ADRC controller are set to 0.02, 135, 25, and 40, respectively. As shown in Figure 10, the slave manipulator can operate the catheter from the initial position to the target position, thereby completing the operation involving the movement of the catheter from the aortic arch to the carotid artery.

Moreover, the target displacements are recorded by the optical mouse sensor in the master manipulator, while the real displacements are recorded by the encoder and grating ruler in circumferential and axial directions, respectively. Furthermore, real-time errors are calculated using Equation (11) as follows:

(11) $$Error_{dis}=Dis_{target}-Dis_{real}$$

where $Dis_{target}$ denotes the target displacement, $Dis_{real}$ denotes the real displacement, and $Error_{dis}$ denotes the error between the last two displacements.

**Figure 11:**
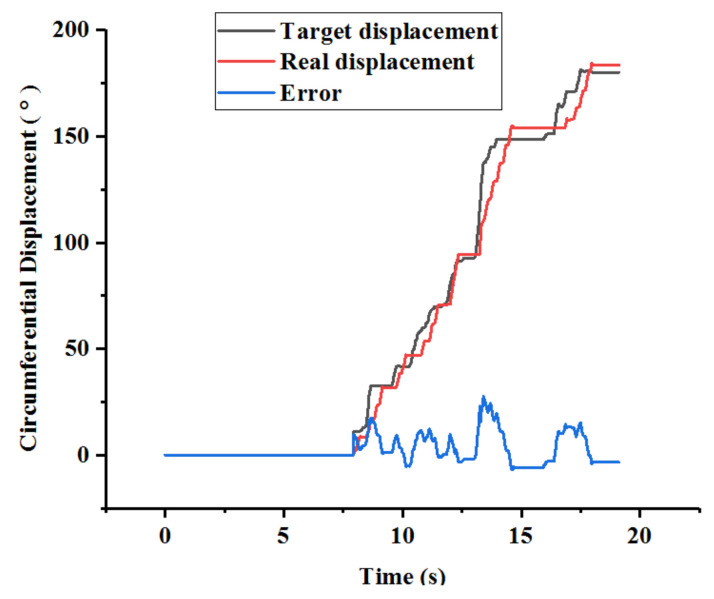
Circumferential displacements during the experiments.

(2) Experimental Results: The operating displacements in the axial and circumferential directions are shown in Figure 10 and Figure 11, respectively. The maximum errors, which refer to the steady-state values, in the axial and circumferential directions are 0.45 mm and 6.67°, respectively.

## 5. Discussions

The results of the evaluation experiments are presented in Figure 10 and Figure 11. As depicted in Figure 10, the operating process is divided into three sub-procedures. In procedure A, the catheter was operated in the axial direction, and the operating displacement was 46.33 mm. In procedure B, the movement direction of the catheter changed via the rotation action, and the circumferential displacement was 53.40°. In procedure C, the catheter was operated from the aortic arch into the carotid artery. Compared with procedures A and B, the displacement increments of procedure C in the axial and circumferential directions were 17.22 mm and 126.90°, respectively. Furthermore, certain errors between the target and real displacements are shown in Figure 10 and Figure 11. The maximum errors, which refer to the steady-state errors, in the axial and circumferential directions were 0.45 mm and 6.67°, respectively. The results show that the proposed system can satisfy the requirements of a safe operation. A previous study [23] showed that if the movement error is less than 2 mm, then the procedure is safe. Moreover, as depicted in Figure 10, there is no overshoot phenomenon, which is crucial in surgery, specifically when treating cerebrovascular diseases. Hence, no overshoot in the axial direction indicates operating safety. Therefore, even if Figure 11 shows an overshoot phenomenon, it does not lead to an accident. Additionally, the real-time errors are unsatisfactory due to the time delay. However, if the master manipulator moves slowly, then the real-time performance can be further improved.

## 6. Conclusions and Future Work

In this study, a novel robotic system using kinetics analysis and an ADRC-based controller is proposed and evaluated. In this system, a master manipulator based on surgeons’ habits and a string-driven slave manipulator were adopted. Additionally, a kinetics analysis and ADRC-based controller was proposed to guarantee the displacement accuracy of the system. The kinetics analysis method is applied to reduce the displacement error influenced by the inner factors of the slave manipulator, and the ADRC-based controller is used to further improve the performance of the system. Based on the experiments using the vascular model, the effectiveness of the kinetics and ADRC-based controller is validated.

In future studies, the proposed method should be tested in clinical experiments. Moreover, the plan to further improve the proposed method is as follows: First, the proposed system should be applied in the EVE module using the slave manipulator to simultaneously operate the catheter/guidewire. Second, experiments should be conducted on living animals to further assess the effectiveness of the proposed method. Finally, a clinical test should be implemented.
